# Borderless collaboration is needed for COVID-19—A disease that knows no borders

**DOI:** 10.1017/ice.2020.162

**Published:** 2020-04-22

**Authors:** Kawthar Mohamed, Eduardo Rodríguez-Román, Farzaneh Rahmani, Hongbo Zhang, Mariya Ivanovska, Sara A. Makka, Musa Joya, Rangarirai Makuku, Md Shahidul Islam, Nesrine Radwan, Laila Rahmah, Rayan Goda, Sunny O. Abarikwu, Mujtaba Shaw, Samaneh Zoghi, Sevan Irtsyan, Irene Ling, Orsolya Cseprekal, Attig-Bahar Faten, Esra Hazar Sayar, Chagajeg Soloukey, Giulia Grancini, Nima Rezaei

**Affiliations:** 1 School of Medicine, Tehran University of Medical Sciences, Tehran, Iran; 2 Universal Scientific Education and Research Network (USERN), Manama, Bahrain; 3 Center for Microbiology and Cell Biology, Instituto Venezolano de Investigaciones Científicas, Caracas, Venezuela; 4 Universal Scientific Education and Research Network (USERN), Caracas, Venezuela; 5 Washington University in St Louis, St Louis, Missouri, United States; 6 Universal Scientific Education and Research Network (USERN), Missouri, United States; 7 Pharmaceutical Sciences Laboratory and Turku Bioscience Centre, Åbo Akademi University, Turku, Finland; 8 Universal Scientific Education and Research Network (USERN), Turku, Finland; 9 Research Center, Department of microbiology and immunology, Medical University, Plovdiv, Bulgaria; 10 Universal Scientific Education and Research Network (USERN), Plovdiv, Bulgaria; 11 Neuroscience Research Center, Faculty of Medical Sciences, Lebanese University, Beirut, Lebanon; 12 Universal Scientific Education and Research Network (USERN), Beirut, Lebanon; 13 Radiology Department, Kabul University of Medical Sciences, Kabul, Afghanistan; 14 Universal Scientific Education and Research Network (USERN), Kabul, Afghanistan; 15 Universal Scientific Education and Research Network (USERN), Harare, Zimbabwe; 16 Department of Tissue Engineering and Applied Cell Sciences, School of Advanced Technologies in Medicine, Tehran University of Medical Sciences, Tehran, Iran; 17 Universal Scientific Education and Research Network (USERN), Dhaka, Bangladesh; 18 Allergy & Immunology Unit, Faculty of Medicine, Ain Shams University, Cairo, Egypt; 19 Universal Scientific Education and Research Network (USERN), Cairo, Egypt; 20 Universal Scientific Education and Research Network (USERN), Jakarta, Indonesia; 21 Clinical Immunology and Allergy Council, Sudanese Society for Clinical Immunology and Allergy, Khartoum, Sudan; 22 Universal Scientific Education and Research Network (USERN), Khartoum, Sudan; 23 Department of Biochemistry, University of Port Harcourt, Choba, Nigeria; 24 Universal Scientific Education and Research Network (USERN), Choba, Nigeria; 25 Universal Scientific Education and Research Network (USERN), Kashmir, India; 26 Ludwig Boltzmann Institute for Rare and Undiagnosed Diseases, Vienna, Austria; 27 Universal Scientific Education and Research Network (USERN), Vienna, Austria; 28 Laboratory Service of Arakbir Medical Center, Yerevan, Armenia; 29 Universal Scientific Education and Research Network (USERN), Yerevan, Armenia; 30 School of Science, Monash University Malasia, Jalan Lagoon Selatan, Selangor Darul Ehsan, Malaysia; 31 Universal Scientific Education and Research Network (USERN), Selangor Darul Ehsan, Malaysia; 32 Department of Transplantation and Surgery, Semmelweis University, Budapest, Hungary; 33 Universal Scientific Education and Research Network (USERN), Budapest, Hungary; 34 Universal Scientific Education and Research Network (USERN), Tunisia, Tunis; 35 University of Rostock, Rostock, Germany; 36 Alenya Training and Research Hospital, Pediatric Allergy Immunology Unit, Alanya, Antalya, Turkey; 37 Universal Scientific Education and Research Network (USERN), Antalya, Turkey; 38 Department of Neuroscience, Erasmus MC, Rotterdam, The Netherlands; 39 Universal Scientific Education and Research Network (USERN), Rotterdam, The Netherlands; 40 Physical Chemistry Unit, Department of Chemistry, University of Pavia, Pavia, Italy; 41 Universal Scientific Education and Research Network (USERN), Pavia, Italy; 42 Universal Scientific Education and Research Network (USERN), Tehran, Iran

*To the Editor*—Coronavirus disease 2019 (COVID-19) has become a global concern among all citizens and governments. Several governments have decided to take drastic actions to combat the spread of the disease, including closure of air, maritime, and land borders as an extreme measures of isolation. However, such measures have not prevented the disease from spreading globally; COVID-19 has already spread to almost all countries and regions, and the World Health Organization (WHO) named it a pandemic on March 11, 2020.^1^ For this reason, some countries have announced a so-called ‘lockdown.’ This protocol includes, but is not limited to, the closure of all nonessential businesses, the obligatory physical isolation of all citizens through quarantine, social distancing for those that do go out of their homes, and public campaigns to encourage both frequent hand washing and refraining from touching the face.

Although these measures seems to be effective in ‘flattening the curve,’ they cannot be applied for a long because they have extreme economic consequences. Vivid examples of such detrimental economic consequences have occurred in Singapore and Hong Kong, where social distancing was applied meticulously. As soon as the measures were withdrawn, a second surge of the disease occurred.^2^ So, what is the solution?

International collaboration seems to be the best tool for curbing the spread of SARS-CoV-2. This virus first hit more wealthy countries where resources and facilities were available, but even these wealthy countries failed to control it. SARS-CoV-2 knows no borders; it has reached every populated region on earth without discrimination. As SARS-CoV-2 spreads to countries with little or no sanitation, low or no hygiene, and few or no hospitals, the conditions could become extreme and dreadful. The prevention of this pending disaster is the role of every country on earth because this pandemic has no borders. Multinational, united efforts are required to end this crisis, which became evident when SARS-CoV-2 spread regardless of border closures. If wealthy countries do not support poor countries in curbing this viral infection, SARS-CoV-2 will find its way back to their countries.

In addition to the humanitarian goals of this collaboration, scientific collaboration is needed as well. The host’s immune system has an important role in the transmission of SARS-CoV-2.^3^ Multiple severe cases within families also suggests a genetic predisposition to COVID-19.^4^ Thus, international collaboration to better understand the disease pathophysiology of COVID-19 is needed. In developed countries, telemedicine provides the opportunity for patients to communicate with physicians remotely via computer.^5^ However, such tools, as well as research, diagnostic kits, and vaccine manufacturers require a huge budget beyond a single country’s financial capability.

This international collaboration should begin before SARS-CoV-2 spreads tragicallly through poor countries. This crisis is growing everywhere on our planet, and if we work together, we might find a solution. For example, a single poor country cannot afford to support test manufacturers. Now the crisis involves mostly rich countries, but what will happen when poor countries without adequate hygiene, sanitation, or well-equipped hospitals and facilities are affected? The consequences could be horrific, with an unimaginable toll.

We should start supporting these poor countries before the virus explodes among them. To refuse to come to their aid is cruel and against humanitarian and moral values. Also, if SARS-CoV-2 spreads uncontrolled, it is more likely to re-emerge in wealthy countries.^6^

The type of collaboration needed has happened before. The smallpox pandemic, a tragedy that killed ~2 million people in 1967, is one example. Nobody believed that the smallpox virus could be stopped, but eventually the goal was achieved, with an intense worldwide collaboration that took ~13 years (1967–1979). The smallpox eradication program was funded by the WHO and 42 other countries. The expense of this accomplishment was merely $112 million in total, or an average of $9 million per year over these 13 years. Some countries spent more individually to stop this pandemic, but their efforts were in vain.^7^ The global effort now needed to stop the COVID-19 pandemic should not be as difficult because of the internet as well as nongovernmental organizations such as the Universal Scientific and Educational Network (USERN), which connects scientists and students from >100 different countries.^8^

In conclusion, we may be wasting important time; a borderless solution for our complex COVID-19 problem could be the best solution overall. In addition to international scientific collaboration, the support of international organizations could help prevent an increase in cases, particularly in countries and regions where COVID-19 is in the early epidemic stage.
